# Current challenges in allergic diseases and computational solutions towards personalized medicine

**DOI:** 10.3389/falgy.2026.1740694

**Published:** 2026-02-05

**Authors:** Nicole Maison, Jimmy Omony

**Affiliations:** 1 Institute of Asthma and Allergy Prevention (IAP), Helmholtz Munich - German Research Center for Environmental Health (GmbH), Munich, Germany; 2 Department of Pulmonary and Allergy, Dr von Hauner Children’s Hospital, Ludwig-Maximilians-University, Munich, Germany; 3 Comprehensive Pneumology Center (CPC-M), Member of the German Center for Lung Research (DZL), Munich, Germany

**Keywords:** allergic diseases, machine learning, personalized medicine, prediction, prevention

## Abstract

Allergic diseases persist as a significant global health concern, profoundly diminishing the quality of life for millions of people across the globe. Allergic diseases exert a growing economic toll worldwide, with prevalence rates rising sharply – now affecting between 10% and 30% of the global population. This upward trend underscores the urgent need for more effective prevention, diagnosis, and management strategies. Rapid urbanization and shifting environmental conditions – particularly those driven by global warming – are increasingly recognized as key contributors to the rising prevalence of allergic diseases worldwide. We examine the current challenges in addressing these complex disorders, from diagnostic limitations to the heterogeneity of clinical presentations. We explore the role of statistical computational tools in predicting allergenicity, offering new avenues for precision medicine in this evolving field.

## Introduction

Allergic diseases – including allergic asthma, atopic dermatitis, allergic rhinitis, and food allergies – represent a significant and growing global health burden affecting individuals across all age groups. These conditions are particularly prevalent among children and adolescents, who bear the greatest disease burden worldwide. Wang et al. (1) and Zemelka-Wiacek et al. (2) provide a comprehensive overview of the immunopathogenesis of allergic diseases, emphasizing the implications for therapeutic strategies. Among the most common allergic disorders, allergic asthma has received the most research attention; however, allergic rhinitis and atopic dermatitis are more widespread globally.

The global prevalence of allergic diseases is estimated to range between 10% and 30%, with substantial regional variation. The highest rates are observed in Western Europe, North America, and increasingly in urban centers of Central Asia (3). Environmental factors such as air pollution, industrialization, and urbanization have been identified as key contributors to the rising incidence of allergies, particularly in pediatric populations. Early-life exposures, such as alterations in the microbiome, also play a critical role in the development of allergic diseases. Additionally, the growing diversity and abundance of food allergens have led to an increase in food allergy prevalence, currently estimated at approximately 5%–10% in children (4, 5).

Recent advances in bioinformatics have enabled deeper insights into mechanisms underlying allergy and facilitated the development of predictive algorithms for allergic disease. Aldakheel (6) underscores the potential of computational approaches to inform future allergology research. Artificial intelligence (AI) in particular, is increasingly employed to improve early detection and risk stratification, especially in the context of pediatric food allergies (7).

Despite notable progress in the diagnosis and management of allergic diseases, several challenges remain unresolved. These include the early identification of individuals with genetic or environmental predispositions, particularly infants and preschool-aged children with food or aeroallergen sensitivities (8). Furthermore, the complexity of allergic disease pathogenesis, diagnostic limitations, phenotypic heterogeneity, and fragmented clinical data continue to hinder effective prevention and personalized treatment.

With the improvement in patient phenotyping and endotyping, there is a personalization impact that enables more precise targeting of therapies like biologics, ICS dosing, and monitoring intensity of allergic diseases like asthma (9) and allergic rhinitis (10). This has been particularly enabled by biomarker discovery for tailored therapy – based on the selection of specific biologic or anti-inflammatory treatments based on genetic/epigenetic signatures (11, 12). For instance, in asthma, there is enhanced adoption of tools predicting ICS treatment response using clinical and environmental data to personalise ICS selection – a development heavily based on advancements in understanding of genetic variation in corticosteroid metabolism for patients. This implies that improved diagnosis and risk stratification results have enhanced diagnostic accuracy (e.g., using feature classification AUC 0.85 approaches like eXtreme Gradient Boosting (XGBoost) using clinical data (9).

### Factors influencing allergic diseases

**Figure 1:**
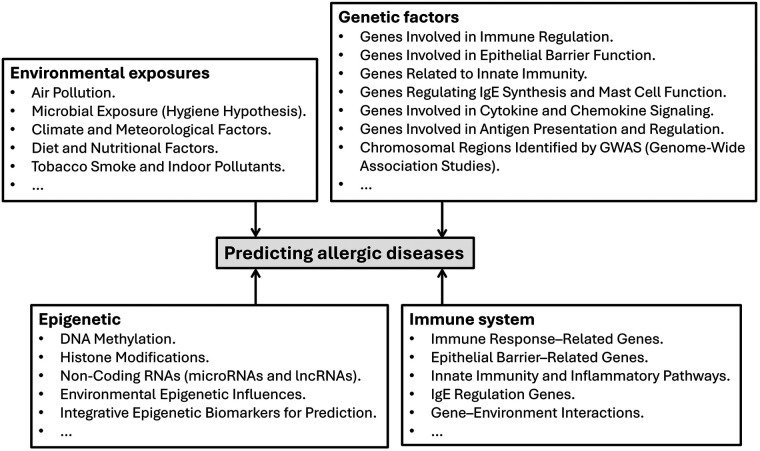
Essential factors used for the prediction of allergic diseases.

Artificial intelligence (AI) is increasingly facilitating the provision and access of information in the field of allergy and immunology (7, 13). The prediction and management of allergic diseases is often complicated by their heterogeneity, which requires personalized and adaptable treatment strategies. Additionally, there is a lack of reliable markers for the phenotyping and prediction of allergic diseases like asthma, allergic rhinitis, food allergies, and atopic dermatitis. There are also increasingly changing environmental and lifestyle factors (14) – especially in the large, populated cities. Figure 1 summarises factors essential for predicting allergic diseases – factors that are essential for integrated bioinformatics analysis. Depending on the platform, various data analytic tools use at least some form of the listed components as input for their allergic disease prediction.

Allergic diseases like allergic asthma, atopic dermatitis, allergic rhinitis, and food allergy, are common in childhood and manifest as heterogeneous diseases (15). The co-occurrence of multiple allergic diseases further complicates the understanding of disease dynamics. Understanding the underlying factors contributing to the allergic diseases in patients is crucial to disease management. Multiple environmental factors, changes in weather and seasonal fluctuations, smoke exposure, and air pollution all contribute to the rise in the number of subjects with allergic conditions. There has, however, been a recent increase in the number of AI tools for the prediction and monitoring of allergic diseases (16). Recently, there is new approaches to treating food allergies, such as the use of oral immunotherapy and biologics to manage patients with immunglobulin E (IgE)-mediated food allergies (17, 18) and more recently, personalized treatment for peanut allergy (19).

### Software for predicting allergic diseases

We collated and summarized tools (or software) used to predict the allergenicity of proteins related to allergic diseases and to forecast allergen exposure. Some of the tools are web-based, and others are standalone analytic tools (Table 1), providing an overview of the different platforms for analysing and handling data on allergenicity and the forecast of allergic diseases, using the factors summarized in Figure 1. In personalized medicine, the use of digital health generally refers to the storage, retrieval, and use of electronic health records (EHRs) in data-driven systems. Digital health covers multiple aspects of health information handling and usage; however, we focus on its use in the context of allergic diseases.

**Table 1:** Overview of example tools/software for allergic disease data-processing, prediction of protein allergenicity, and analysis.

Tool/software	Description
AllergyPred (20)	a web server for allergen prediction, analysis of protein sequences, structure, features and immunogenic properties to assess allergenicity risks
ALLERDET (21)	A novel web app for prediction of protein allergenicity, detect new allergens from amino acid sequence in FASTA format – tool for discovering new allergens
Digital Health Systems (22)	The present and future of digital health (storage and use of electronic health records), digital medicine, and digital therapeutics for allergic diseases
ChAIPred (23)	A Web Server for Prediction of Allergenicity of Chemical Compounds, predicting chemical allergens, designing chemical analogs with desired allergenicity
AllerCatPro 2.0 (24)	a web server for predicting protein allergenicity potential
AllerTool (25)	a web server for predicting allergenicity andallergic cross-reactivity in proteins
AlgPred, AlgPred 2.0 (26)	Web-server, an improved method for predicting allergenic proteins and mapping of immunoglobulin E (IgE) epitopes
iAller (27)	Computational prediction of allergenic proteins based on multi-feature fusion, predicting the allergenicity of protein efficiency
Allerdictor (28)	fast allergen prediction using text classification techniques, a sequence-based allergen prediction tool that models protein sequences.
SEP-AlgPro (29)	An efficient allergen prediction tool utilizing traditional machine learning and deep learning techniques with protein language model features
CTPAD (30)	an interactive web application for comprehensive transcriptomic profiling in allergic diseases
SDAP 2.0 (31)	The updated Structural Database of Allergenic Proteins (SDAP 2.0) provides 3D models for allergens and incorporated bioinformatics tools
AllerTOP, AllerTOP v.2 (32)	AllerTOP – a server for in silico prediction of allergens
Peanut-Allergic predictor (33)	Open Access Online Calculator Predicts Peanut-Allergic Reactions With High Accuracy
SORTALLER (34)	SORTALLER: predicting allergens using substantially optimized algorithm on allergen family featured peptides
Allermatch™ (35)	Allermatch™, a webtool for the prediction of potential allergenicity according to current FAO/WHO Codex alimentarius guidelines, queries amino acid sequence prediction of allergenicity of proteins
Alg-MFDL (36)	Alg-MFDL: A multi-feature deep learning framework for allergenic proteins prediction
CTPAD (30)	an interactive web application for comprehensive transcriptomic profiling in allergic diseases, R shiny App
AllergyMonitor (37)	Digital technologies for an improved management of respiratory allergic diseases: 10 years of clinical studies using an online platform for patients and physicians, short-term prediction of allergic symptoms
AirCare (an App)	Usable on mobile devices, https://getaircare.com/
AirRater (an App)	Usable on mobile devices, https://airrater.org/
Zyrtec's AllergyCast (an App)	Usable on mobile devices, https://www.zyrtec.com/allergy-forecast

A pending challenge is that many of the tools/software developed based on basic research have not been integrated (or translated) for use in clinical practice. Hopefully, within the next decade, some of these tools can be used in clinical diagnosis and prediction of allergic diseases; thus, moving science to the clinic and improving patient quality of life.

Overall, allergenicity prediction tools/software improve allergy prediction. They help in anticipating allergic responses by analyzing the biological and chemical features of a given compound. The tools improve the predictive accuracy and strengthen early-stage allergy risk assessment – further minimizing the reliance on trial-and-error testing in clinical practice. Allergenicity prediction tools aid risk stratification and enable early warning, mitigating the risk for allergic diseases. They aid prediction of the likelihood of a substance triggering an allergic response. Allergenicity tools also enable comparison of structural and sequence homology between known allergens and candidate substances. Hence, the tools help in the prediction of whether individuals allergic to one source (e.g., an aeroallergen or food proteins) may also react to related allergens.

We have also identified and summarized software, mobile apps for allergen and pollen prediction – aimed at preventing allergic diseases. These tools enable control and prevention of allergen symptoms. We grouped these tools into three categories: (i) research/clinical apps, (ii) developer Application Programming Interface (APIs)/data sources, and (iii) consumer apps (Table 2). We list platforms and specify key features that make each useful for preventing allergy symptoms, e.g., by taking medication in advance. Although not the subject of this mini review, we note that most app tools are run on the iOS/Android platform, and the specifics of the application can be obtained from the corresponding references.

**Table 2:** Overview of software, mobile apps for allergens and pollen prediction.

Category	Description of application
Research/clinically validated apps	**PollDi** (from Helmholtz Munich, University of Augsburg) – an evidence-backed digital intervention — app for grass-pollen allergy sufferers (pollen forecasts, air-quality data, symptom diary). PollDi reduced symptoms and resulted in improved quality of life in a trial (PollDi).
Developer APIs & data sources (for building prediction tools or integrating pollen layers)	**BreezoMeter API** — commercial, high-resolution pollen and air-quality APIs for embedding forecasts into third-party apps (https://www.docs.breezometer.com).
	**National Allergy Bureau (AAAI/NAB)** — authoritative measured pollen and mold counts (map and station data) — appropriate for “ground truth” local counts in NAB stations (https://www.pollen.aaaai.org).
	**https://www.Pollen.com** **services** — established pollen forecasts and tools (some apps/auto integrations use https://www.Pollen.com).
Consumer mobile apps (for people who want forecasts, alerts and symptom tracking)	**Allergy Plus (****https://www.Pollen.com****/Allergy Alert family)** — location-based 5-day pollen forecasts, allergy diary and alerts (US/global coverage via https://www.Pollen.com data, AllergyPlus).
	**Allergy Pollen Count/Allergy Pollen Count (NAB-based apps)** — apps display National Allergy Bureau (NAB) certified sampler counts — best applicable for lab-measured local values rather than modelled forecasts (AllergyPollenCount).
	**AccuWeather (Allergy/Allergy forecast section)** — pollen indices for tree/grass/ragweed + day-by-day allergy risk guidance inside the mainstream weather app. Useful if you already use AccuWeather (AccuWeather).
	**klarify (Pollen, Allergy app)** — pollen, weather, air quality, and daily symptom logging, and a 3-day forecast; focuses on helping you track triggers over time (klarify).
	**ZYRTEC® AllergyCast®** — personalized “allergy impact” score plus pollen counts and symptom tracker. Aids anticipation of how a patient will feel following medication (GooglePlay).
	**BreezoMeter (Air Quality, Pollen)** — real-time pollen and air-quality forecasts, hyperlocal modelling and exposure advice; widely used by apps and platforms for pollen layers, and is best applicable for combined AQ + pollen risk (BreezoMeter).

### Sensitivity and specificity of SPT and IgE tests in the diagnosis of allergic disease

The currently available computational tools (both open source/commercial) and routinely used clinical assays for diagnosing and predicting allergic diseases vary in their sensitivity and specificity. This variability depends on the biomarkers used, as well as the underlying computational algorithms. Clinical assays like the skin prick tests (SPT) and the serum-specific IgE tests (e.g., ImmunoCAP) have high sensitivity (approximately up to 90%). The SPT is used to confirm the presence of sensitization in IgE-mediated allergic diseases (38, 39). Across respiratory and food allergy diagnostics, multiple studies indicate that SPT and serum-sIgE each have specific advantages, with their performance varying according to allergen type, extract quality, patient characteristics, and clinical context. While these two tests may provide partially overlapping diagnostic information, particularly in food allergy, they can still offer complementary insights in certain settings. The component-resolved diagnostics (CRD) represent a distinct analytical approach that provides additional, and in some cases, more refined information. However, it should be considered separately from the conventional extract-based SPT and specific IgE framework discussed here.

Wagner and Rudert (40) investigated the sensitivity and specificity of SPTs of standardized allergen extracts based on SPT for the diagnosis of IgE-mediated respiratory allergies. They assessed a large, multi-center study that compared SPT results with benchmark methods, including specific IgE measurements. They observed that: (i) sensitivity of the SPT increases with higher extract concentrations, while the specificity decreases accordingly, and (ii) there is a broad consensus between the SPT and specific IgE tests, although the performance metrics fundamentally differ between allergens. This demonstrates that the sensitivity and specificity of the SPT vary depending on the conditions, and that trade-offs exist between them*.*

Choi et al. (41), studying house dust mite (HDM) allergy in asthma, found SPT to be more sensitive (81%) but less specific (52%) than sIgE. The sIgE showed lower sensitivity (67%) but higher specificity (71%), supporting the use of SPT for screening and sIgE for confirmation. Alimuddin et al. (42) further corroborated this paradigm in a cohort of 101 patients (with respiratory allergy), showing that SPT detected broader sensitization profiles, while sIgE achieved higher specificity (up to ∼90%) for clinically relevant allergens. Chauveau et al. (43) reported moderate agreement between SPT and sIgE in a large cohort of children with asthma or hay fever, with SPT demonstrating higher specificity than sIgE and similar sensitivity, reinforcing the value of combined testing in paediatric allergy.

In food allergy, the limitations of both tests are more pronounced. Foong et al. (39) reviewed approaches of improving the diagnostic accuracy of food allergy, noting that the SPT and sIgE tests have high sensitivity and negative predictive value. Foong et al. also highlight the limitations of both SPT and serum IgE tests – including aspects of their specificity, which is often low for clinical practice. Ta et al. evaluated SPT and sIgE against double-blind, placebo-controlled food challenges and found that SPT wheal sizes over 5 mm had high sensitivity (∼91%) but low specificity (∼50%) for clinical reactions. Combining SPT and sIgE improved specificity at the cost of sensitivity, and neither test correlated well with reaction severity, underscoring their limited predictive value when used alone. These findings align with broader evidence that food allergy diagnostics are highly allergen-dependent and benefit from multimodal approaches.

Meta-analyses provide consolidated evidence supporting these trends. Rigionni et al. (44), analysing 149 studies encompassing over 24,000 patients, showed that SPT using fresh food extracts achieved high sensitivity (90%–94%), making it well suited for screening, while component-resolved sIgE testing (e.g., Ara h2) demonstrated very high specificity (∼92%–95%), offering strong confirmatory value. For allergic rhinitis, Nevis et al. (45) reported a pooled SPT sensitivity of ∼85% and a specificity of ∼77%, though with wide variability across studies. Meta-analyses using other food allergies similarly resulted in variable sensitivities (68%–92%) and specificities (58%–88%) for SPT. Extract-based sIgE often shows lower specificity when using non-optimized thresholds.

Overall, the evidence indicates that the diagnostic performance of SPT and sIgE varies by allergen, patient age, and clinical setting. For food allergy, combining SPT, sIgE, and component-resolved diagnostics improves diagnostic accuracy. For respiratory allergy, SPT – particularly with standardized extracts – serves as a sensitive screening tool, while sIgE refines diagnostic specificity when used in combination, thus improving its clinical relevance. Current allergy diagnostics show substantial variability in sensitivity and specificity, which limits their value for personalized medicine. SPTs and serum-sIgE often achieve high sensitivity but inconsistent specificity, with wide disease-dependent ranges. The use of AI aims to improve both metrics. However, multiple concurrent exposures to aero- and food-allergens complicate computational refinement of SPT and sIgE accuracy. These inconsistencies pose a major modeling challenge, as personalized approaches require robust algorithms to distinguish true clinical allergy from sensitization and integrate heterogeneous biomarkers.

## Discussion

The prevalence of allergic diseases is on the rise. Predicting individuals who will develop an allergy is complicated, partly due to the clinically heterogeneous nature of the diseases. Knowing which medication (or treatments) might work for an individual requires an integrated approach – using noisy data from clinical records, data from environmental exposure, data from immune biomarkers, and the now popular multi-omics approaches (46, 47). We discuss principal clinical and computational challenges in allergic disease diagnosis and management.

Owing to the heterogeneity of allergic diseases (and unclear endotypes – often poorly characterized), there is still a practice to use the same label to group many mechanistic pathways (e.g., asthma and eczema) – even though the patients differ in disease history, trigger of disease, and associated comorbidities (and lifestyles) (48, 49). It is conceivable that the allergic disease underlying subtype (so-called endotypes) requires refinement in definition (1). To date, characterizing endotypes remains a challenge – even though tremendous progress is being made to resolve it.

There is a lack of validated and easily accessible biomarkers for the prediction (and diagnosis) of allergic diseases (50). Personalized medical approaches require a multi-omic data integration approach. There is still a lack of reliably predictive biomarkers – ones that are specific and scalable to a wide range of allergic diseases. With the promise of high-dimensional and high-resolution omics data (e.g., genomics, transcriptomics, proteomics, metabolomics) comes the high financial and computational costs. There is complexity in standardizing assay batches, sometimes with noisy data that requires specially tailored software for data processing. Recent articles highlight both the promise and the practical obstacles of multi-omics in allergy (51).

Prediction of allergic diseases is also hindered by poor data quality, poor data entry, and documentation inconsistencies in electronic health records EHRs. Even though clinical records are the primary data source for prediction, allergy documentation in EHRs is often inaccurate, incomplete, or heterogeneous (free-text notes, wrong labels, historical entries that are not reconciled). Missingness, miscoding, and interoperability problems distort model training and create unsafe clinical decision prompts (false alerts or missed allergies). Several reviews and position statements argue that in the field of allergy, EHRs need a drastic structural reform before large-scale predictive tools can be trusted.

It is well known that the exposome and dynamic environmental exposures to allergies are shaped by lifetime environmental exposures, including air pollution, microbiome shifts, diet, chemicals, and climate effects (52). Measuring the exposome is conceptually straightforward but technically and analytically fraught; however, it is often expensive, with equipment that needs to be installed and frequently monitored in various locations in different cities of developed countries.

The heterogeneity and thus complexity of predicting and managing allergic disease requires a collaborative approach from multidisciplinary research fields such as immunology, environmental science, and bioinformatics. Robust statistical models and data-integrative bioinformatics approaches are required to generate signals from often noisy data. Genetic and environmental data need to be utilized to enhance the predictive performance of models for allergic diseases. EHRs, if available, need to be exploited to provide extra information for personalized predictive models. Though not the core of our discussion here, there is a need to globally strengthen and enact life-impacting environmental policies to reduce the prevalence of allergic diseases.

## Conclusion

Tremendous progress has been made in the diagnosis, prevention, and management of allergic diseases – particularly in developed countries. However, there is still a vast difference in access to medical facilities for diagnosing allergic diseases. The use of AI and machine learning approaches in the prediction and diagnosis of allergic diseases remains limited, not only in developed countries but also in major conditions such as asthma, atopic dermatitis, and allergic rhinitis. With increasing costs associated with access to and treatment of allergic diseases, there is a need to develop early-life diagnostic kits and expand outreach programs to ensure the population receives proper diagnosis and treatment for allergic diseases.
